# Genetic characterization and description of *Leishmania* (*Leishmania*) *ellisi* sp. nov.: a new human-infecting species from the USA

**DOI:** 10.1007/s00436-023-08034-8

**Published:** 2023-12-15

**Authors:** Sarah G. H. Sapp, Ross Low, Gabriela Nine, Fernanda S. Nascimento, Yvonne Qvarnstrom, Joel L. N. Barratt

**Affiliations:** 1 Division of Parasitic Diseases and Malaria, Centers for Disease Control and Prevention, Atlanta, GA USA; 2 Oak Ridge Institute of Science and Education, Oak Ridge, TN USA; 3 Association of Public Health Laboratories, Silver Spring, MD USA

**Keywords:** *Leishmania*, Leishmaniasis, United States, *Leishmania ellisi*, *Leishmaniamexicana* complex, Arizona

## Abstract

In a 2018 report, an unusual case of cutaneous leishmaniasis was described in a 72-year-old female patient residing in Arizona, United States of America (USA). Preliminary analysis of the 18S rDNA and glyceraldehyde-3-phosphate dehydrogenase genes supported the conclusion that the *Leishmania* strain (strain 218-L139) isolated from this case was a novel species, though a complete taxonomic description was not provided. Identification of *Leishmania* at the species level is critical for clinical management and epidemiologic investigations so it is important that novel human-infecting species are characterized taxonomically and assigned a unique scientific name compliant with the ICZN code. Therefore, we sought to provide a complete taxonomic description of *Leishmania* strain 218-L139. Phylogenetic analysis of several nuclear loci and partial maxicircle genome sequences supported its position within the subgenus *Leishmania* and further clarified the distinctness of this new species. Morphological characterization of cultured promastigotes and amastigotes from the original case material is also provided. Thus, we conclude that *Leishmania* (*Leishmania*) *ellisi* is a new cause of autochthonous cutaneous leishmaniasis in the USA.

**Supplementary information:**

The online version contains supplementary material available at 10.1007/s00436-023-08034-8.

## Introduction

The genus *Leishmania* is a diverse and complicated taxon, comprised of approximately 30 species across 4 subgenera. Historically, identification and taxonomic assignments were based on extrinsic factors (e.g., vector identity, clinical presentation) and biochemical characteristics via isoenzyme profiling. With the advent of molecular tools, our ability to identify and classify *Leishmania* strains has improved, with particular benefits in laboratory diagnosis of leishmaniasis. It follows that a consequence of this revolution is that sequence analysis of causative agents of leishmaniasis may reveal unexpected results, fundamentally changing our understanding of geographic range, clinical features, and host specificity of established species in addition to revealing novel species. Examples include the recently described *Leishmania* (*Mundinia*) *chancei* from Ghana (Kwakye-Nuako et al. 2023) and *Leishmania* (*Mundinia*) *orientalis* originally isolated in Thailand (Jariyapan et al. 2018).

In 2018, an unusual case of cutaneous leishmaniasis (CL) was reported in a 72-year-old female patient residing in the United States of America (USA), within the State of Arizona (de Almeida et al. 2021). The patient reported no history of international travel and had a history of granulomatosis with polyangiitis, chronic sinusitis, chronic kidney disease, and pulmonary coccidioidomycosis. The case was referred to the US Centers for Disease Control and Prevention (CDC) for *Leishmania* species identification by PCR and Sanger sequencing of the Internal transcribed spacer 2 (ITS2) locus (GenBank accession: MT764332.1). The sequencing results from this investigation suggested that the isolate (henceforth *Leishmania* strain 218-L139) was not a match to any known species described at the time. Genome sequencing (Illumina MiSeq) was conducted and several gene sequences were extracted from this Illumina data for phylogenetic analysis, including the 18S rRNA gene and glyceraldehyde-3-phosphate dehydrogenase gene (de Almeida et al. 2021). Rudimentary phylogenies constructed from these loci supported that strain 218-L139 shared a recent evolutionary relationship with *Leishmania* (*Leishmania*) *mexicana* (de Almeida et al. 2021), though was not a member of this species. The authors of the original report concluded that “classification of this parasite cannot be conclusively determined based solely on [the] genetic evidence observed,” so a species description was not provided (de Almeida et al. 2021).

Identification of *Leishmania* at the species level is critical for clinical management and epidemiologic investigations (de Almeida et al. 2021), so it is important that novel human-infecting *Leishmania* species are characterized taxonomically and assigned a scientific name if deemed appropriate. Without an appropriate taxonomic description, discussions of subsequent cases or investigations into the new organism can be cumbersome and may create chaos in the scientific literature. In the past, “new” *Leishmania* parasites that had been characterized molecularly, clinically, and morphologically, but left unnamed, were designated subsequently in the scientific literature by names qualifying as *nomina nuda* (names applied to organisms in the absence of a complete taxonomic description, which violates the code set by the International Commission on Zoological Nomenclature (ICZN)) and/or simply by a strain designation. This pitfall is well-illustrated in the confusion plaguing the literature on cases of CL caused by species within the subgenus *Mundina* between the 1990s and 2018 (Anuntasomboon et al. 2022; Barratt et al. 2017; Sereno 2019).

The present study sought to provide a complete taxonomic description of *Leishmania* strain 218-L139 by reexamining the taxonomic relationship between it and other *Leishmania* species. We present phylogenetic analyses including several nuclear genes and the maxicircle genome. For completeness of the description, we also include a morphological description of cultured promastigotes and in situ amastigotes as they appear in the touch preparation slides prepared as part of the original case description of strain 218-L139 (de Almeida et al. 2021). Based on this analysis, we confirm that *Leishmania* strain 218-L139 is indeed a novel species, and assign it the name *Leishmania* (*Leishmania*) *ellisi* sp. nov. (Kinetoplastida: Trypanosomatidae) in honor of eminent Professor John T. Ellis, whose career in parasitology began with a PhD dissertation on the molecular phylogeny of *Leishmania*.

## Methods

### Parasite cultivation and morphological characterization

Promastigotes of *Leishmania* strain 218-L139 were cultured to generate material for morphologic description. Cryopreserved stocks of cultures grown from the original case biopsy material were thawed and propagated by standard methods, including in axenic complete RPMI (10% fetal bovine serum) with and without gentamicin (0.02 mg/mL) and in cultures containing Vero E-6 cells (de Almeida et al. 2021; Lawrence and Sapp 2021). We also sought to generate sequence data for *Zelonia australiensis*, a close relative of *Leishmania* that is immediately basal to the dixenous members of the Leishmaniinae subfamily, and therefore serves as an important reference taxon for inclusion in phylogenetic reconstructions of this group (Barratt et al. 2017). *Zelonia australiensis* promastigotes were cultivated axenically in the liquid media M3, as described previously (Barratt et al. 2017). Briefly, a 2 g/L solution of powdered bovine hemoglobin (BD BBL, NJ, USA) was autoclaved at 121 °C for 15 min. Once cooled, 500 mL of this hemoglobin solution was added aseptically to 500 mL of complete M199; pH 7, with 10% heat-inactivated horse serum (Bovogen Biologicals, Victoria, Australia) and single-strength penicillin–streptomycin, sterile filtered. Finally, 10 mL of IsoVitaleX (BD BBL) was aseptically added to the solution to a final volume of 1.01 L of complete M3 media.

Giemsa-stained smears were prepared from this cultured material and promastigotes were measured at 1000 × magnification under oil emersion microscopy using Olympus CellSens Standard software. Giemsa-stained touch preparations from the original leishmaniasis case patient (de Almeida et al. 2021) were reexamined and amastigote measurements were taken as described above. Measurements of unstained promastigotes in culture were also taken at 400 × magnification under DIC microscopy.

## Phylogenetic analysis

### Reference sequences for target loci

Two phylogenies were constructed to investigate the evolutionary relationship between *Leishmania* strain 218-L139 and other *Leishmania* species. One phylogenetic tree was constructed from a concatenated sequence including five nuclear loci: the 18S and 28S rRNA genes, Casein Kinase II α chain (CKIIα), glycosomal glyceraldehyde 3-phosphate dehydrogenase (gGAPDH), and RNA polymerase II largest subunit (RPOIILS) genes. A second tree was constructed from partial maxicircle genome sequences. Reference sequences of these loci were extracted from published genome sequences of various trypanosomatids using BLASTN searches and/or were obtained from the GenBank nucleotide database by manual searches. GenBank accession numbers for each locus included in these phylogenetic reconstructions are provided in Supp. File S1. Illumina MiSeq data were generated previously for *Leishmania* strain 218-L139 (de Almeida et al. 2021) (data available in NCBI under BioSample ID: PRJNA1028282). Accurate sequences of the 18S rRNA, 28S rRNA genes, CKIIα, GAPDH, and RPOIILS loci and the maxicircle were generated from this *Leishmania* strain 218-L139 Illumina MiSeq data using the methods below. Illumina HiSeq data were generated for *Zelonia australiensis* (BioSample ID: PRJNA1028289) and sequences of these target loci were extracted from the *Z. australiensis* HiSeq data, as described below.

### Illumina data generation and analysis

DNA was extracted from a log-phase culture of *Zelonia australiensis* using a DNeasy Blood & Tissue DNA extraction kit (Qiagen, Hilden, Germany). The resultant DNA extract was sent to the Australian Genome Research Facility (AGRF, https://www.agrf.org.au/) for sequencing on the Illumina HiSeq platform. Accurate consensus sequences of the 18S rRNA and 28S rRNA genes, CKIIα, GAPDH, RPOIILS, and the maxicircle genome were generated from the Illumina MiSeq data previously generated for *Leishmania* strain 218-L139 and from the *Z. australiensis* HiSeq data by aligning the reads to published reference sequences from other Leishmaniinae parasites.

Briefly, the forward and reverse reads were trimmed for quality using the bbduk tool provided as part of the BBMap toolkit (version 38.73) (Bushnell 2014) using the following user-defined parameters: minlen = 50, qtrim = rl, trimq = 30, ktrim = r, k = 23, mink = 11, hdist = 1. The resulting trimmed reads were merged using bbmerge (also part of the BBMap toolkit) with the following parameters: rem k = 62, extend2 = 50 ecct. The resulting merged and unmerged reads were mapped to a set of *Leishmania* or *Zelonia* references sequences of the target loci (GenBank accessions; CKIIα: CALT01000026.1:16,800–18800, 18S rDNA: CP029526.1:1,013,476–1015665, 28S rRNA: JQ648649.1, GAPDH: DQ092549.1, RPOIILS: AF126254.2, maxicircle: MK514116.1), using bwa aligner (default parameters). The fastq reads mapping to each reference sequence were extracted from the resulting sam files using samtools (Li et al. 2009). The mapped fastq files (one for *Z. australiensis* and one for *Leishmania* strain 218-L139) were imported into Geneious Prime (Biomatters LTD, Auckland, New Zealand: https://www.geneious.com). Read mapping was performed in Geneious Prime once again using the “Map to reference” function with the following parameters: maximum gap of 10% per read, maximum gap size of 15 bases, minimum overlap of 25 bases, minimum overlap similarity of 80%, maximum 20% mismatches per read, and a maximum of 4 ambiguities. All other values were set to default. The resultant alignment files were viewed manually in Geneious to assess the quality of mapping. After mapping accuracy was confirmed visually, a consensus sequence was generated for each locus for *Z. australiensis* and *Leishmania* strain 218-L139.

### Phylogenetic tree construction

For the concatenated gene tree, 33 trypanosomatid taxa were represented, including members of each of the four *Leishmania* subgenera—i.e., *Leishmania*, *Sauroleishmania*, *Viannia*, and *Mundinia* (GenBank and/or TriTrypDB accession numbers in Supp. file S1, Tab A). Sequences were aligned using MUSCLE 3.8.4 and the alignments were manually curated using AliView v1.28. The five nuclear sequences were concatenated to a final length of 6577 positions. Model estimation, maximum likelihood phylogenies, and bootstrapping (1000 replicates) were estimated using IQ-tree 1.6.12. Bayesian inference and posterior probability values were calculated with MrBayes 3.2.7a.

A large fragment of the maxicircle genome was extracted from published genome sequences of 67 trypanosomatid taxa for construction of our second tree (GenBank and/or TriTrypDB accession numbers in Supp. file S1, Tab B). Complete maxicircle genome sequences could not be obtained for all 67 taxa of interest, though a large fragment of the maxicircle was obtained for all of them. These partial maxicircle sequences were aligned to a final length of 9471 positions. Model estimation, maximum likelihood phylogenies, and bootstrapping (1000 replicates) were estimated using IQ-tree 1.6.12. Bayesian inference and posterior probability values were calculated with MrBayes 3.2.7a.

## Results

### Cultivation and morphological characterization

**Fig. 1:**
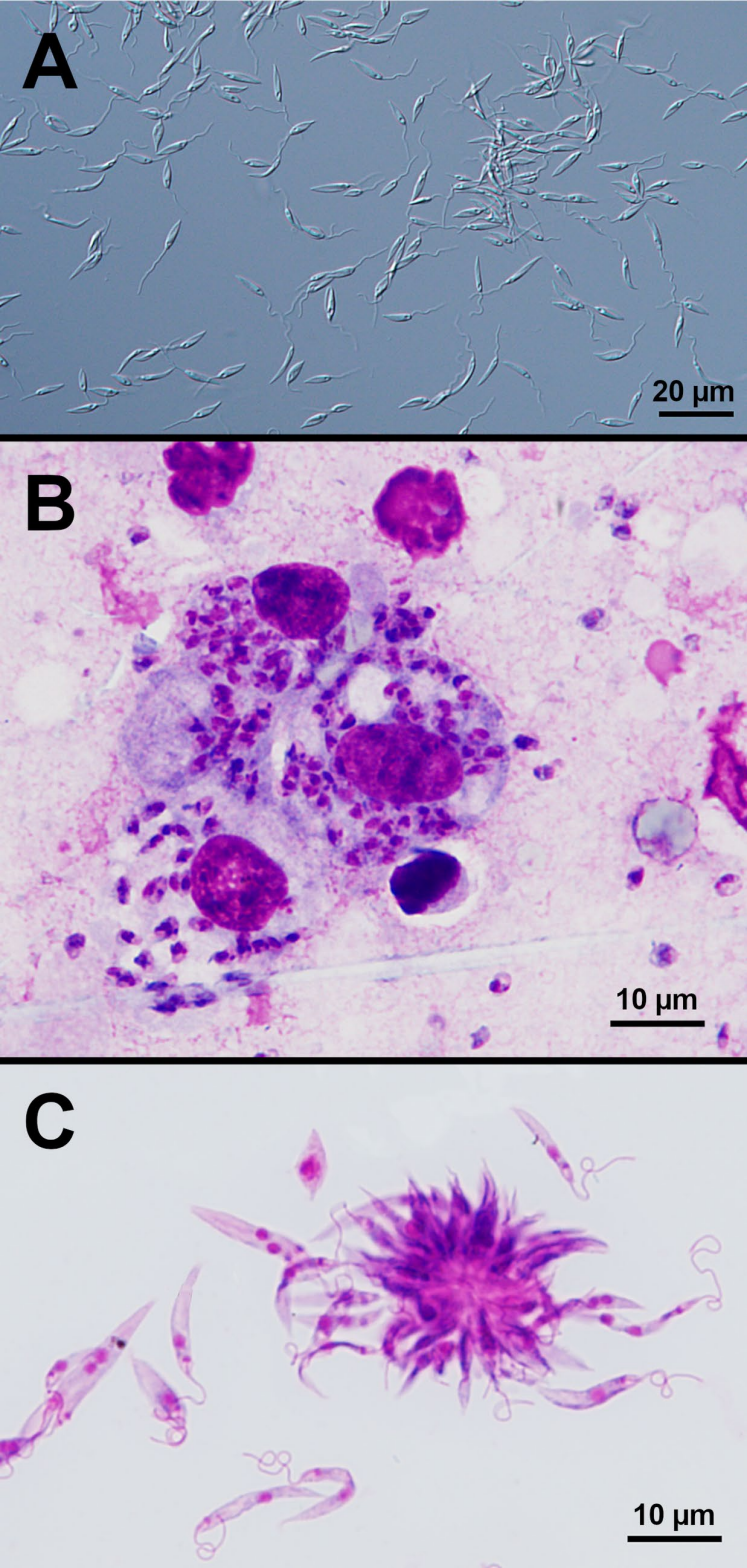
Morphology of *Leishmania* strain 218-L139 promastigotes and amastigotes. Cultured *Leishmania* strain 218-L139 promastigotes as observed under DIC microscopy following growth in complete RPMI culture medium without gentamicin (**A**). Giemsa-stained touch preparation made from a cutaneous lesion, showing *Leishmania* strain 218-L139 amastigotes inside a mononuclear phagocyte in addition to free amastigotes (**B**). Giemsa-stained smear of cultured promastigotes of *Leishmania* strain 218-L139 grown in gentamicin-free complete RPMI medium (**C**)

Live promastigotes (Fig. 1A) ranged from 6.1 to 13.4 µm (average 10.1 µm) in length including the flagellum; promastigotes in Giemsa-stained preparations measured 5.7 to 15.4 µm (average 9.6 µm) (Fig. 1C). Amastigotes observed in Giemsa-stained touch preparations ranged from 2.7 to 3.2 µm (average 2.7 µm) on the longest axis (Fig. 1B).

Although this study was not designed to characterize culture growth characteristics, it was observed that promastigotes grew vigorously only under the culture conditions which did not include gentamicin. In cultures containing gentamicin (0.02 mg/mL), promastigotes developed morphological aberrations (rounded forms and small flagella) over the course of about 1 week; such cultures became completely inviable by around 10 days. In contrast, cultures without gentamicin appeared healthy on day 9; motile cells with typical promastigote morphology and some rosettes were evident indicating replication (Supp. file S2).

### Phylogenetic analysis

**Fig. 2:**
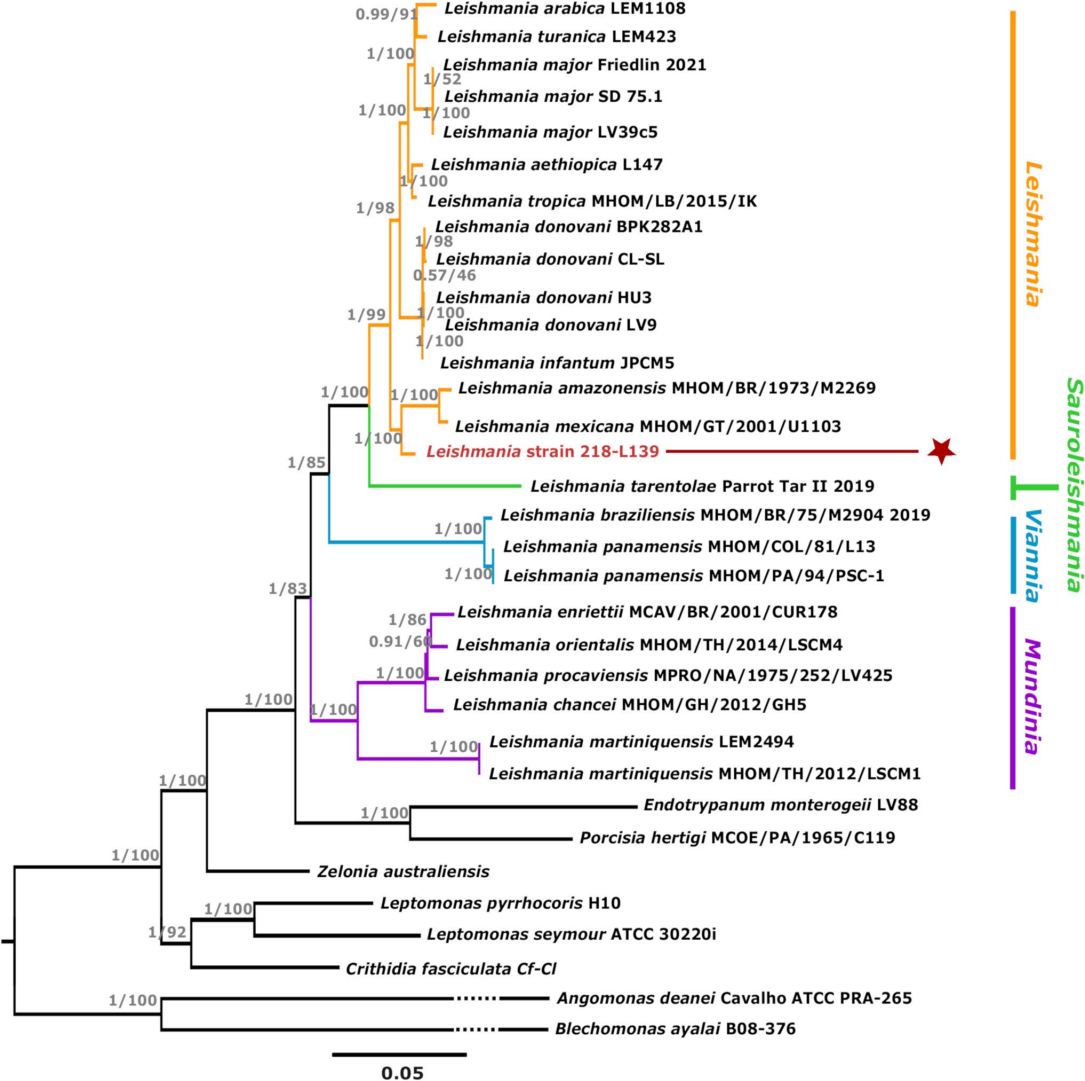
Phylogenetic tree demonstrating the relative position of *Leishmania* sp. strain 218-L139 based on a concatenated sequence comprising several nuclear loci. Phylogenetic tree demonstrating the relative position of *Leishmania* strain 218-L139 (brown star) based on alignment of a concatenated sequence comprising the 18S rDNA, 28S rDNA, CKIIα, GAPDH, and RPOIILS nuclear sequences. The alignment contains 6577 positions from 33 taxa. The tree was built using maximum likelihood (1000 bootstrap replicates) and Bayesian inference using GTR + I + G substitution model. Posterior probability values and bootstrap support are reported for each node (pp/bs). The tree was rooted with the *Angomonas*/*Blechomonas* clade. The relative position of each of the *Leishmania* subgenera is shown. Branch lengths within the *Angomonas*/*Blechomonas* clade were truncated for readability. The scale bar represents the number of substitutions per site.

**Fig. 3:**
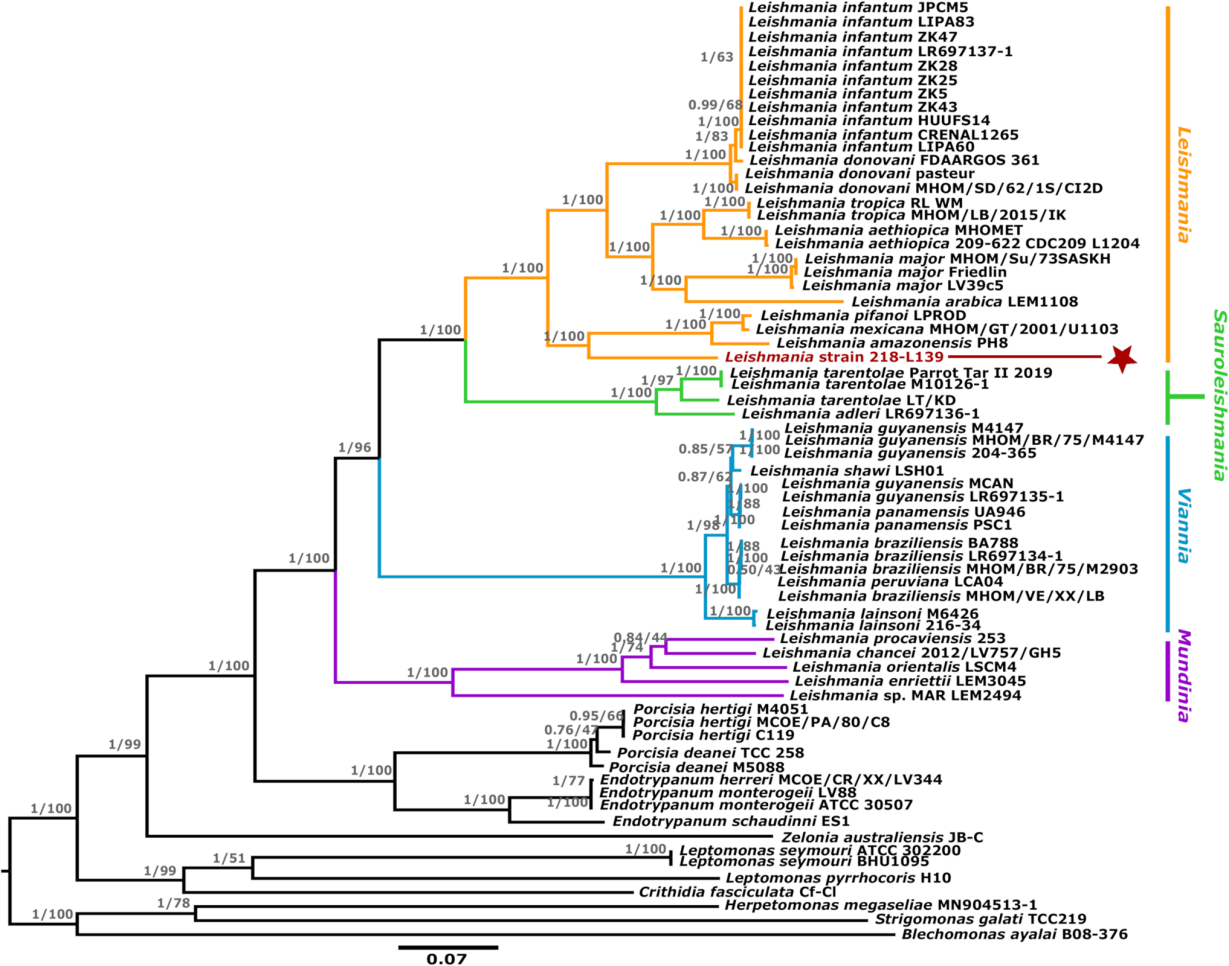
Phylogenetic tree demonstrating the relative position of *Leishmania* strain 218-L139 based on partial maxicircle genome sequences. Phylogenetic tree demonstrating the relative position of *Leishmania* strain 218-L139 (brown star) based on alignment of partial maxicircle genome sequences. The alignment contains 9471 positions from 67 taxa. The tree was built using maximum likelihood (1000 bootstrap replicates) and the TVM + F + I + G4 model, and Bayesian inference using the GTR + I + G model where the proportion of invariable sites was set to 0.1. Posterior probability values and bootstrap support are reported for each node (pp/bs). The tree was rooted with the *Strigomonas*/*Blechomonas*/*Herpetomonas* clade. The relative position of each *Leishmania* subgenus is shown to the right of the corresponding clades (*Leishmania*, *Sauroleishmania*, *Viannia*, *Mundinia*). The scale bar represents the number of substitutions per site

Consensus sequences were generated for *Z. australiensis* and *Leishmania* strain 218-L139 for the five nuclear loci and the partial maxicircle region selected for phylogenetic analysis (GenBank accessions in Supp. file S1). Maximum likelihood trees generated from both the concatenated nuclear (CN) and maxicircle (MAX) sequences (6577 and 9471 positions, respectively) were robust, with strong bootstrap support (Fig. 2, Fig. 3). Both trees reflected monophyly for the four *Leishmania* subgenera and supported that *Leishmania* strain 218-L139 is a member of the *Leishmania* subgenus, closely related to *Leishmania mexicana* and *Leishmania amazonensis* (posterior probability of 1 and 100% bootstrap support, both trees). The genetic distance between *L. mexicana* and *L. amazonensis* computed from our CN and MAX alignments were 0.0076 and 0.053, respectively (Supp. file S1, Tabs C & D), while *L. mexicana* and *Leishmania* strain 218-L139 were separated by much larger genetic distances of 0.021 and 0.15, respectively. Similarly, strain 218-L139 and *L. amazonensis* were separated by distances of 0.022 (CN) and 0.15 (MAX). For comparison, genetic distances between *L. braziliensis* and *L. panamensis* were 0.006 (CN) and 0.014 (MAX), and the distances separating *L. tropica* and *L. major* (CN:0.011, MAX:0.13), *L. donovani* and *L. infantum* (CN:0.0009, MAX:0.0074), and *L. aethiopica* and *L. major* (CN:0.015, MAX:0.13) were also less than the genetic distance separating strain 218-L139 from other members of the *L. mexicana* complex. As such, *Leishmania* strain 218-L139 was confirmed as being markedly distinct from other members of the *L. mexicana* complex, warranting a species-level taxonomic distinction. We subsequently assigned *Leishmania* strain 218-L139 the name *Leishmania* (*Leishmania*) *ellisi* sp. nov. in honor of parasitologist and Emeritus Professor J. T. Ellis, whose career in parasitology began with a PhD dissertation on the molecular phylogeny of *Leishmania*.

## Taxonomic summary

### *Leishmania* (*Leishmania*) *ellisi* sp. nov. Barratt, Sapp, and Qvarnstrom 2023

- Synonyms: *Leishmania* sp. strain 218-L139, *Leishmania* sp. strain Arizona.
- Taxonomy: Phylum Euglenozoa (Cavalier-Smith, 1981) emend. (Cavalier-Smith, 2016); class Kinetoplastea (Honigberg, 1963) emend. (Vickerman, 1976); subclass Metakinetoplastina (Vickerman, 2004); order Trypanosomatida (Kent, 1880); family Trypanosomatidae (Doflein, 1901); subfamily Leishmaniinae (Maslov and Lukes 2012) emend. (Shaw, Camargo and Teixeira 2016); genus *Leishmania* (Ross, 1903); subgenus *Leishmania* (Shaw, Camargo and Teixeira, 2016); species *Leishmania* (*Leishmania*) *ellisi* (Sapp, Qvarnstrom, Barratt, 2023).
- Type host: Humans, *Homo sapiens*. Vector is currently unknown.
- Type locality: Arizona, USA.
- Type material: Hapanotype, cryopreserved promastigotes stored in liquid nitrogen and original touch impression slide archived at the Centers for Disease Control and Prevention, USA.
- Strain designation: MHOM/US/2018/L139.
- Morphology: Cultured promastigotes are an average of 10.1 ± 2.2 µm in length (including the flagellum), ranging between 6.1 and 13.4 µm in length. Amastigotes in fixed and stained touch preparations are an average of 2.7 ± 0.26 µm on the longest axis, with a range of 2.7 to 3.2 µm. In Giemsa-stained preparations made from cultures, promastigotes are an average of 9.6 ± 3.4 µm in length (flagellum included), with a range of 5.7 to 15.4 µm.
- Etymology: *Leishmania* (*Leishmania*) *ellisi* is named in honor of parasitologist and Emeritus Professor J. T. Ellis, whose career in parasitology began with a PhD dissertation on the molecular phylogeny of *Leishmania* species.
- Diagnosis: *Leishmania* (*Leishmania*) *ellisi* is most closely related to *Leishmania* (*Leishmania*) *mexicana* and *Leishmania* (*Leishmania*) *amazonensis*. In Giemsa-stained preparations, its amastigotes are typical to those of other *Leishmania* species, and its promastigotes are typical in appearance compared to other *Leishmania* (*Leishmania*) species. *Leishmania* (*Leishmania*) *ellisi* possesses unique sequences at its 18S rRNA, 28S rRNA, Casein Kinase II α chain, Glyceraldehyde 3-phosphate dehydrogenase, and the RNA polymerase II largest subunit genes, and has a unique maxicircle sequence; sequences for these loci are publicly available under GenBank accession numbers OR689570, OR689569, OR695082, OR695080, OR695081, and OR701861, respectively.

## Discussion

This study provides the first taxonomic description of *Leishmania* sp. strain 218-L139—now *Leishmania* (*Leishmania*) *ellisi* which appears to be a member of the *L. mexicana* complex and is a cause of cutaneous leishmaniasis in the USA. While numerous cases of autochthonous human leishmaniasis have been reported from Texas and Oklahoma (Clarke et al. 2013; Fumer 1990; Gustafson et al. 1985; McHugh 2010; McHugh et al. 1996; Nelson et al. 1985; Shaw et al. 1976), and once from North Dakota (Douvoyiannis et al. 2014), this represents the first case of CL from Arizona (de Almeida et al. 2021). Nearly all autochthonous US leishmaniasis cases have been attributed to *L.* (*L*.) *mexicana* (with one exception; a member of *L. donovani/infantum* in the North Dakota case). However, species identities from these US cases were typically derived from clinical characteristics and/or isoenzyme analysis, methods lacking in terms of resolving power relative to modern sequencing techniques (Fumer 1990; Gustafson et al. 1985; McHugh et al. 1996; Nelson et al. 1985; Shaw et al. 1976). This raises the question of whether this case from Arizona (de Almeida et al. 2021) truly represents the first recorded case of leishmaniasis attributable to *L.* (*L.*) *ellisi*. Retrospective sequencing analysis of cultured material from past cases will be necessary to illuminate the range of species causing autochthonous CL within the USA, particularly in reference to the diversity within the *L. mexicana* complex.

Clinical traits observed in other members of the *L. mexicana* complex are consistent with what is currently known regarding *L.* (*L.*) *ellisi* sp. nov. For example, CL is the typical manifestation observed in association with the *L. mexicana* complex, where *L.* (*L.*) *mexicana* typically manifests as localized cutaneous leishmaniasis (LCL, also referred to as chiclero’s ulcer when the ear is affected specifically) though will occasionally manifest as diffuse cutaneous leishmaniasis (DCL), which is rarer and more severe (Andrade-Narvaez et al. 2001; Canche-Pool et al. 2022; Fernandez-Figueroa et al. 2012). *Leishmania* (*L.*) *amazonensis* also causes LCL and DCL, though seemingly has a greater propensity for causing DCL than *L. mexicana* (Franca-Costa et al. 2012). The index case of *L.* (*L.*) *ellisi* also manifested as LCL in an immunocompetent person, which resolved spontaneously (de Almeida et al. 2021).

We observed that gentamicin inhibits *L. ellisi* growth in vitro. This follows reports from previous authors describing gentamicin as an attenuation agent for multiple *Leishmania* species (Daneshvar et al. 2003, 2009). Gentamicin has also been used as a topical treatment for cutaneous disease, in combination with paromomycin (Sosa et al. 2019). In our experience, *Leishmania* isolates vary in their apparent tolerance to gentamicin in culture media; further experimentation is required to characterize these differences and associated mechanisms.

Further exploration of the diversity within the subgenus *Leishmania* in vertebrate hosts and sandflies in North America is required to reveal the identity of reservoir(s) and vectors of *L.* (*L*.) *ellisi.* Given that other members of the *L. mexicana* complex are generally host-promiscuous, it is likely that *L.* (*L.*) *ellisi* may be a zoonotic species with a primary mammalian host other than humans (de Almeida et al. 2021). Veterinary cases of *L.* (*L.*) *mexicana* infection have been reported in the US Southwest for decades, namely in cats and dogs (Craig et al. 1986; Hopke et al. 2021; Kipp et al. 2016). Rodents also become naturally infected with *L.* (*L.*) *mexicana*, so likely serve as important reservoirs (Grogl et al. 1991; Kipp et al. 2016; Sosa-Bibiano et al. 2022). Bats have been identified as possible reservoirs of *L*. (*L.*) *mexicana* in parts of Mexico (Berzunza-Cruz et al. 2015), and in Brazil, *L.* (*L.*) *amazonensis* has been detected in black rats (Caldart et al. 2017), cats (Nascimento et al. 2022), and dogs (Tolezano et al. 2007). Data on potential vectors of *L.* (*L.*) *mexicana* complex parasites is relatively scarce, though natural vectors of *L.* (*L.*) *mexicana* may include *Psathyromyia shannoni*, *Lutzomyia cruciate* (Perez-Blas et al. 2022), and *Lutzomyia longipalpis* (da Costa et al. 2019). Vectors of *L.* (*L.*) *amazonensis* also largely remain a mystery, though *Lutzomyia cruzi* (de Oliveira et al. 2017), *Lu. longipalpis* (Carvalho-Silva et al. 2022), and *Lutzomyia flaviscutellata* (Carvalho et al. 2015) represent potential vectors. These phlebotomine species may represent potential vectors of *L.* (*L.*) *ellisi*, and screening them for the presence of *L.* (*L.*) *ellisi* DNA is indicated as a starting point in the search for its vector/s.

As the morphology of *Leishmania* species is generally conserved and therefore taxonomically uninformative, and isoenzyme analysis is considered deprecated, characterization of novel *Leishmania* species is now almost entirely based on molecular phylogenetic approaches (Kaufer et al. 2017; Votypka et al. 2015). However, developing a sufficiently robust *Leishmania* phylogeny for taxonomic purposes requires careful selection of taxa included in the phylogenetic reconstruction: one must ensure that these taxa cover the evolutionary “spectrum” of recognized Leishmaniinae species. For instance, representatives of each of the four *Leishmania* subgenera (i.e., *Leishmania*, *Sauroleishmania*, *Viannia*, and *Mundinia*) should be included in a phylogenetic reconstruction, in addition to members of the genera *Porcisia* and *Endotrypanum*. This will help ensure that a novel taxon falling within the Leishmaniinae subfamily is assigned to the appropriate monophyletic subgenus. Inclusion of *Zelonia* spp*.*, which are basal to all dixenous Leishmaniinae (Barratt et al. 2017), may improve bootstrap support at some internal nodes. Finally, inclusion of other monoxenous Leishmaniinae taxa such as *Leptomonas* and selecting an appropriate outgroup will improve the robustness of phylogenetic reconstructions for this group (Kaufer et al. 2019). The selected outgroup should not be too evolutionarily distant or branches within the ingroup may be compressed, confounding the trees’ interpretation; this phenomenon may be observed when using *Trypanosoma* or *Pseudotrypanosoma* to root phylogenetic reconstructions aiming to characterize a novel taxon within the Leishmaniinae (Pearson et al. 2013). In our experience, the monoxenous non-Leishmaniid trypanosomatid genera *Blechomonas* and/or *Herpetomonas* are useful outgroup candidates that generally facilitate the construction of informative Leishmaniinae phylogenies.

The choice of loci included in a phylogenetic reconstruction is also of paramount importance. Kaufer et al. describe the coding region of the maxicircle as a “superior” taxonomic marker for the *Leishmaniinae* (Kaufer et al. 2019, 2020), and the present study sought to utilize part of this sequence to support the present taxonomic summary on that basis. The phylogeny in Fig. 2 was constructed from a concatenated sequence comprising the 18S and 28S rRNA genes, CKIIα, GAPDH, and the RPOIILS genes; these nuclear-encoded loci are considered phylogenetically informative and most have been used previously to define novel taxa within the *Leishmaniinae* (Barratt et al. 2017; Kostygov et al. 2016; Votypka et al. 2015). These loci evolve at different rates such that single-gene phylogenies may present a slightly different evolutionary picture from one another. For instance, the rRNA genes are among the most highly conserved loci in nature (Isenbarger et al. 2008); a consequence of them being functionally constrained meaning they evolve very slowly. As such, rRNA sequences may be useful for separating distantly related taxa, but can result in compression of branches among closely related taxa. Concatenating rRNA sequences to sequences from more rapidly evolving genes such as gGAPDH improves taxonomic resolution by countering this compression effect. As noted previously (Espinosa et al. 2018; Kaufer et al. 2020; Votypka et al. 2015), phylogenies generated from a concatenated sequence comprised of several loci will generally result in superior phylogenies, and by concatenating sequences of loci that vary in their evolutionary rate, resultant phylogenies should provide a more accurate representation of the evolutionary relationships among the taxa included in a phylogenetic reconstruction.

Espinosa et al. (2018) suggest that in an ideal scenario, multiple isolates of a new candidate *Leishmaniinae* taxon should be sequenced prior to making new taxonomic assignments. However, this is not always practical for organisms that are rarely encountered. For clinically significant “novel” taxa such as *L.* (*L.*) *ellisi* which was identified from a geographic area where autochthonous leishmaniasis is not uncommon (i.e., Southwestern USA), the need to provide a clear taxonomic diagnosis that distinguishes it from other causes of leishmaniasis in the region (namely *L.* (*L.*) *mexicana*) necessitates the present taxonomic description. Furthermore, it should also be highlighted that the long delay in assigning a name to *L.* (*M.*) *macropodum* via means of a formal taxonomic description (despite the existence of reports describing host, vector, and nucleotide data that supported such a description (Dougall et al. 2009, 2011; Rose et al. 2004)) led to the use of the “*Leishmania* australiensis” *nomen nudum* widely within the scientific literature, necessitating a re-description to establish a species name (now *L.* (*M*.) *macropodum* Barratt, Kaufer, and Ellis, 2017) (Barratt et al. 2017). Further taxonomic issues related to the *Mundinia* have also occurred as a direct result of not performing a timely and complete taxonomic description. “*Leishmania siamensis*” is an example of another *nomen nudum* that was applied incorrectly to CL cases from disparate parts of the world and from varying hosts, which were later confirmed as being caused by *L.* (*M*.) *martiniquensis* and *L.* (*M*.) *orientalis*. The presence of this “*L. siamensis*” *nomen nudum* creates confusion in reviews and retrospective analyses of literature and sequences in which this name was used, and authors of current reports still often rely on bulky parenthetical clarification (e.g., “*L.* (*M*.) *orientalis* (formerly named *L.* (*M*.) *siamensis*”)) to overcome this (Anuntasomboon et al. 2022)*.* Importantly, our referring to the delayed taxonomic summaries above is not a condonation of rushed taxonomic summaries based on limited data; these delayed descriptions occurred in the context of having sufficient data to warrant an earlier taxonomic description—particularly in the case of *L.* (*M.*) *macropodum*. Instead, these issues are highlighted to emphasize that the existence of viable parasite cultures, whole genome sequence data, morphological descriptions, phylogenetic analyses, and a clinical case report amply support our taxonomic summary of *L.* (*L.*) *ellisi* and favor not delaying its description any further to prevent similar taxonomic issues arising later.

Regardless of the concerns described above, Espinosa et al. (2018) make a valid point, and to demonstrate that a taxonomic distinction is indeed appropriate for *L.* (*L.*) *ellisi*, we endeavored to provide ample phylogenetic support derived from numerous loci. As we point out above, the genetic distances between *L.* (*L.*) *ellisi* and *L.* (*L.*) *mexicana* and/or *L.* (*L.*) *amazonensis* (its nearest sister taxa) are far greater than the genetic distances computed between *L.* (*L.*) *mexicana* and *L.* (*L.*) *amazonensis*. Similarly, *L.* (*L.*) *ellisi* is more disparate from other members of the *L. mexicana* complex compared to *L. braziliensis* versus *L. panamensis* or *L. major* versus *L. tropica*; these pairs are less genetically disparate from one another yet have been recognized as separate species for decades. If we apply a current taxonomic “yardstick” standard that already exists for the Leishmaniinae to the present study, then *L.* (*L.*) *ellisi* is certainly sufficiently distinct to warrant a taxonomic distinction.

To further confirm the distinct specific status of *L. ellisi*, we performed additional analyses to compare it to two lesser-known members of the *L. mexicana* complex—*Leshmania* (*L.*) *waltoni* and *Leishmania* (*L.*) *venezuelensis*—however this was not included in our broader phylogenetic analysis due to a paucity of sequence data for those species. After mapping whole genome sequencing reads from *L.* (*L.*) *ellisi* to partial (486 base pair fragments) RPOIILS sequences from *L*. (*L*.) *waltoni* (GenBank: KM555334) and *L*. (*L*.) *venezuelensis* (GenBank: KM555334), we observed that *L.* (*L.*) *ellisi* differed from these two parasites by 12 SNPs at this locus. In contrast, *L*. (*L*.) *waltoni* and *L*. (*L*.) *venezuelensis* differed from *L.* (*L.*) *mexicana* by only 2 to 3 SNPs at this locus. Thus, in applying this same taxonomic “yardstick” rationale as established for Leishmaniinae, our data support that *L.* (*L.*) *ellisi* is sufficiently different from *L*. (*L*.) *waltoni* and *L*. (*L*.) *venezuelensis* to warrant its taxonomic separation.

Despite the important role that phylogenetics plays in defining new trypanosomatid taxa, Votypka et al. (2015) suggest that an ideal system of trypanosomatid taxonomy should combine the traditional system that incorporates life-cycle, morphologic, clinical, and host/vector information, with modern phylogenetic methods. While the present taxonomic description is missing some of these elements (i.e., information on vectors and host range is not available), we capture most of them, and our molecular data provide support in favor of a taxonomic distinction between *L.* (*L.*) *ellisi* and its nearest relatives. Votypka et al. also point out that “[the] name-bearing type is the keystone element for classification, systematics, nomenclature, or taxonomy since it is considered the reference specimen defining a species”, and highlight the importance of maintaining hapantotypes and other type material as part of a complete taxonomic description (Votypka et al. 2015). In line with this, in addition to the sequence data presented, type material in the form of DNA extracts, viable cryopreserved promastigotes, and stained touch preparations (preserved slides) containing *L.* (*L.*) *ellisi* promastigotes is stored in the CDC’s parasitology reference laboratory. This material therefore completes the taxonomic description of *Leishmania* (*Leishmania*) *ellisi* sp. nov., a new member of the *Leishmania mexicana* complex and a cause of cutaneous leishmaniasis in the USA.

### Supplementary information

Below is the link to the electronic supplementary material.

Supplementary file1 (XLSX 65 KB)

Supplementary file2 (AVI 2126 KB)

## Data Availability

All sequence data referenced in this manuscript are publicly available. Whole genome shotgun data for *Leishmania ellisi* are available in the NCBI database under BioSample ID PRJNA1028282. Whole genome shotgun data for *Zelonia australiensis* are available under BioSample ID PRJNA1028289. *Leishmania ellisi* sequences generated in this study are available in the GenBank nucleotide database under accession numbers OR689569, OR689570, OR695080, OR695081, OR695082, and OR701861. *Zelonia australiensis* sequences generated in this study are available in the GenBank nucleotide database under accession numbers OR689568, OR689571, OR695077, OR695078, and OR695079.
